# Advanced SPR-Based Biosensors for Potential Use in Cancer Detection: A Theoretical Approach

**DOI:** 10.3390/s25092685

**Published:** 2025-04-24

**Authors:** Talia Tene, Fabian Arias Arias, Darío Fernando Guamán-Lozada, María Augusta Guadalupe Alcoser, Lala Gahramanli, Cristian Vacacela Gomez, Stefano Bellucci

**Affiliations:** 1 Department of Chemistry, Universidad Técnica Particular de Loja, Loja 110160, Ecuador; 2 Department of Chemistry and Chemical Technologies, University of Calabria, Via P. Bucci, Cubo 9 15D, 87036 Arcavacata di Rende, Italy; 3 Facultad de Ciencias, Escuela Superior Politécnica de Chimborazo (ESPOCH), Riobamba 060155, Ecuador; 4 Nano Research Laboratory, Excellent Center, Baku State University, AZ1148 Baku, Azerbaijan; 5 Chemical Physics of Nanomaterials, Physics Department, Baku State University, AZ1148 Baku, Azerbaijan; 6 INFN-Laboratori Nazionali di Frascati, Via E. Fermi 54, 00044 Frascati, Italy

**Keywords:** surface plasmon theory, cancer, Kretschmann configuration, transfer matrix method, silicon nitride, black phosphorus

## Abstract

This study presents a numerical investigation of surface plasmon resonance (SPR) sensors based on multilayer configurations incorporating BK7, silver, silicon nitride (Si_3_N_4_), and black phosphorus (BP). Using the transfer matrix method, the optical performance of four architectures was evaluated under refractive index perturbations consistent with values reported in prior theoretical and experimental studies. The sensor response was characterized through metrics such as angular sensitivity, resonance shift, full width at half maximum, attenuation, and derived figures including detection accuracy and limit of detection. Parametric optimization was performed for the thickness of each functional layer to enhance sensing performance. Among all configurations, those incorporating both Si_3_N_4_ and BP demonstrated the highest angular sensitivity, reaching up to 394.46°/RIU. These enhancements were accompanied by increased attenuation and spectral broadening, revealing trade-offs in sensor design. The results, based entirely on numerical modeling, provide a comparative framework for guiding SPR sensor optimization under idealized optical conditions.

## 1. Introduction

Biosensors are analytical platforms designed to detect chemical or biological targets through specific physicochemical interactions transduced into measurable signals [1]. Among the various transduction modalities—electrochemical [2], piezoelectric [3], and optical [4,5]—surface plasmon resonance (SPR) has established itself as a powerful mechanism due to its label-free nature and capacity for high-sensitivity detection of refractive index changes at the metal–dielectric interface [6]. SPR sensors operate by exciting surface plasmon waves at the boundary between a noble metal and a dielectric medium when p-polarized light is incident under total internal reflection [7]. Any perturbation in the optical environment adjacent to the interface induces a shift in the resonance angle, offering a sensitive route for probing local refractive index variations. Such sensitivity has led to the widespread application of SPR systems in molecular recognition, environmental monitoring, and biochemical analysis [8].

Ongoing advances in plasmonic device engineering have produced a broad range of SPR sensor configurations, including multilayer designs and nanoscale patterning strategies aimed at enhancing field confinement and spectral resolution. Roh et al. [9] discussed how nanostructures such as gratings, nanohole arrays, and waveguide-coupled architectures improve light–matter interaction by localizing the plasmonic fields. In a related approach, Huraiya et al. [10] introduced a bowtie-shaped photonic crystal fiber sensor exhibiting increased amplitude sensitivity and a reduced full width at half maximum (FWHM), facilitated by optimized modal dispersion in the sensing region. Geometry-induced plasmonic tuning has also been explored by Zaman and Hesselink [11], who studied C-shaped nano-apertures in metallic films. Their work demonstrated that the transmission and field enhancement in such structures are highly dependent on the polarization of incident light and the asymmetry of the aperture geometry. By combining full-field electromagnetic simulations with an equivalent circuit model, they provided a framework for linking physical geometry to inductive–capacitive behavior in the optical domain. These findings emphasize the potential of polarization-sensitive nanostructures to fine-tune the optical response of SPR platforms beyond conventional designs.

Apart from geometric optimization, the incorporation of advanced materials into SPR sensors continues to be a key driver of performance improvements. Two-dimensional (2D) materials such as black phosphorus (BP) [12] exhibit high carrier mobility, a tunable bandgap, and anisotropic optical properties, making them promising candidates for plasmonic enhancement layers [13]. In parallel, dielectric materials like silicon nitride (Si_3_N_4_) offer advantages such as high refractive index, low propagation loss in the visible range, and compatibility with CMOS fabrication techniques [14]. When combined with noble metals such as silver (Ag) or gold (Au) [15,16], these materials can lead to SPR structures with enhanced sensitivity, narrower resonance linewidths, and improved long-term stability [17].

The conventional Kretschmann configuration remains a widely adopted platform for SPR excitation due to its structural simplicity and compatibility with theoretical modeling [18]. A BK7 glass prism is typically used to couple incident light into surface plasmon modes at the metal interface [19]. The reflectance behavior of the system under various optical and geometrical parameters can be accurately described using the transfer matrix method (TMM) [20], which enables layer-by-layer computation of electromagnetic field continuity across interfaces. In numerical simulations of SPR systems, variations in the refractive index of the sensing medium are commonly employed to represent changes induced by the presence of analytes [21]. While this simplification captures the first-order optical response, it does not account for more complex factors associated with biological specimens, such as scattering, absorption, morphology, and binding dynamics. The optical behavior of real biological samples—including cells and tissues—is governed by a combination of refractive, absorptive, and structural properties, which are not fully captured by the scalar refractive index alone [22]. As such, modeled systems based purely on refractive index shifts should be interpreted as general perturbative scenarios rather than direct representations of specific biological states.

Alternative techniques capable of probing individual micro-objects with higher fidelity have also been proposed. One such method, known as spectral tweezers, was introduced by Zaman et al. [23]. This approach combines optoelectronic tweezers (OETs) with high-resolution spectroscopic interrogation to trap and analyze single particles. By integrating spatial confinement with optical characterization, spectral tweezers enable label-free spectral profiling of biological samples at the single-cell level, offering a valuable complement to SPR-based methods.

The present work investigates the numerical behavior of a multilayer SPR sensor composed of silver, black phosphorus, and silicon nitride films in a BK7 prism-based Kretschmann configuration. The objective is to evaluate how small refractive index changes (1.33–1.41) [24] in the sensing medium affect the sensor’s performance across different material arrangements. All simulations are performed using the transfer matrix method. Key metrics such as resonance angle shift, sensitivity, FWHM, and quality factor (QF) are used to assess sensitivity under idealized conditions. This study does not attempt to model real biological interactions but instead provides a theoretical proof-of-concept aimed at optimizing SPR sensor designs for future experimental implementation.

## 2. Methodology

### 2.1. Numerical Framework

The full modeling details are given in Ref. [25]. The total reflection of the *Nth*-layer model can be described as follows:

(1) $$R={\left|\frac{\left(M_{11}+M_{12}\;q_{N}\right)q_{1}-\left(M_{21}+M_{22}\;q_{N}\right)}{\left(M_{11}+M_{12}\;q_{N}\right)q_{1}+\left(M_{21}+M_{22}\;q_{N}\right)}\right|}^{2}$$

By Equation (1), the so-called SPR curve can be obtained, giving access to the peak position, full-width half maximum (FWHM), and attenuation percentage. Then, the performance sensing metrics of the proposed SPR biosensors are denoted as follows:

- The first parameter is the sensitivity enhancement regarding the baseline sensors after/before pathogen/molecule adsorption, denoted as follows (Equation (2)):

(2) $$∆S_{RI}^{after}=\frac{\left(S_{RI}^{after}-S_{RI}^{before}\right)}{S_{RI}^{before}}$$

- Then, the sensitivity to the refractive index change can be denoted as follows (Equation (3)):

(3) $$S_{RI}=\frac{∆\theta }{∆n}$$

Here, ∆θ represents the angle shift variation in degrees and ∆n represents the refractive index variation in dimensionless.

- The detection accuracy (DA) can be expressed in terms of ∆θ and FWHM (in degrees) of the SPR curve, as follows (Equation (4)):

(4) $$DA=\frac{∆\theta }{FWHM}$$

- The quality factor (QF) can be expressed in terms of $S_{RI}$ and FWHM, as follows (Equation (5)):

(5) $$QF=\frac{S_{RI}}{FWHM}$$

- The figure of merit (FoM) can be expressed as follows (Equation (6)):

(6) $$FoM=\frac{S_{RI}(1-R_{min})}{FWHM}$$

Here, $R_{min}$ represents the lowest normalized reflection value of the SPR curve.

- The limit of fetection (LoD) can be calculated as follows (Equation (7)):

(7) $$LoD=\frac{∆n}{∆\theta }\times 0.005{}^{\circ}$$

- The comprehensive sensitivity factor (CSF) ratio can be calculated (Equation (8)) [26,27]:

(8) $$CSF=\frac{S_{RI}\times (R_{max}-R_{min})}{FWHM}$$

$R_{max}$ represents the maximum reflectance before resonance deep. All computations are carried out with a data sampling of $5\times 10^{4}$ points.

### 2.2. Systems Under Investigations

**Figure 1:**
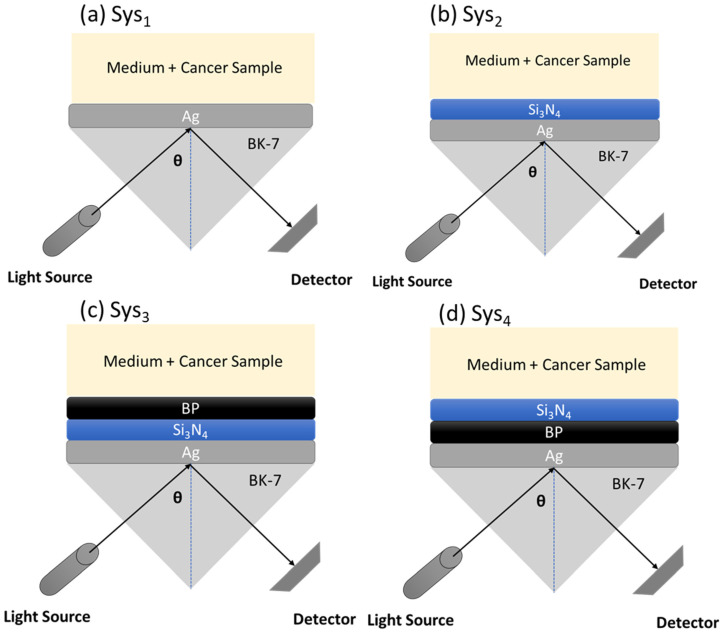
Schematic illustration of the different systems under investigation. (**a**) Sys_1_, (**b**) Sys_2_, (**c**) Sys_3_, and (**d**) Sys_4_.

To begin, this study evaluates the performance of four multilayer configurations based on the Kretschmann prism-coupled SPR setup. All configurations share a common base structure consisting of a BK7 glass prism, a thin silver (Ag) layer as the plasmonic medium, and a semi-infinite sensing region. Variations are introduced through the inclusion and ordering of additional dielectric and two-dimensional (2D) materials: silicon nitride (Si_3_N_4_) and black phosphorus (BP). The interaction of p-polarized light with these multilayer stacks is modeled to study the angular reflectance behavior under small variations in the refractive index of the external medium. A schematic illustration of the systems under investigation is presented in Figure 1, with full system identifiers provided in Table S1. The corresponding refractive indices and layer thicknesses are summarized in Table S2.

In Figure 1a, Sys_1_ represents the fundamental SPR configuration comprising BK7 (prism), a 55.0 nm Ag layer, and a homogeneous external dielectric medium with a refractive index of 1.349 [24]. This configuration serves as a baseline system to study plasmonic excitation without any enhancement layer. In Figure 1b, Sys_2_ introduces a 5.0 nm Si_3_N_4_ layer between the silver and the external medium. Silicon nitride (*n* = 2.0394) is used to enhance field confinement and facilitate coupling between the surface plasmon wave and the dielectric region [13]. Its inclusion has been shown to improve resonance stability in previous optical and photonic structures. In Figure 1c, Sys_3_ extends Sys_2_ by incorporating a 0.53 nm BP monolayer between the Si_3_N_4_ and the external medium. Black phosphorus, with a refractive index of 3.5 + 0.01i at the working wavelength [27], introduces anisotropic optical behavior and strong light–matter interaction, contributing to improved sensitivity and field localization. In Figure 1d, Sys_4_ reverses the position of the BP and Si_3_N_4_ layers compared to Sys_3_. Here, BP is directly interfaced with the Ag layer, followed by Si_3_N_4_, and then the dielectric medium. This arrangement is designed to assess the impact of layer order on resonance sharpness and sensing depth.

To further emphasize, all systems utilize BK7 glass as the base coupling prism (*n* = 1.5151 at 633 nm [17]), which enables the excitation of surface plasmons under total internal reflection. Silver is used as the plasmonic metal due to its favorable real and imaginary permittivity components at optical frequencies (*n* = 0.056253 + 4.2760i at 633 nm [26]), supporting efficient surface plasmon propagation.

The refractive index of the external sensing medium is varied in simulations across the range of 1.33 to 1.41, with reference values of 1.335 for phosphate-buffered saline (PBS) [16] and 1.349 as a test value for a perturbed environment [24]. These variations are intended to simulate generalized optical shifts in the surrounding medium rather than to represent specific biological structures. As such, the configurations are analyzed under idealized refractive index perturbations, consistent with the theoretical scope of this study and without attributing diagnostic specificity.

## 3. Results and Discussion

### 3.1. Systems Under Consideration

**Figure 2:**
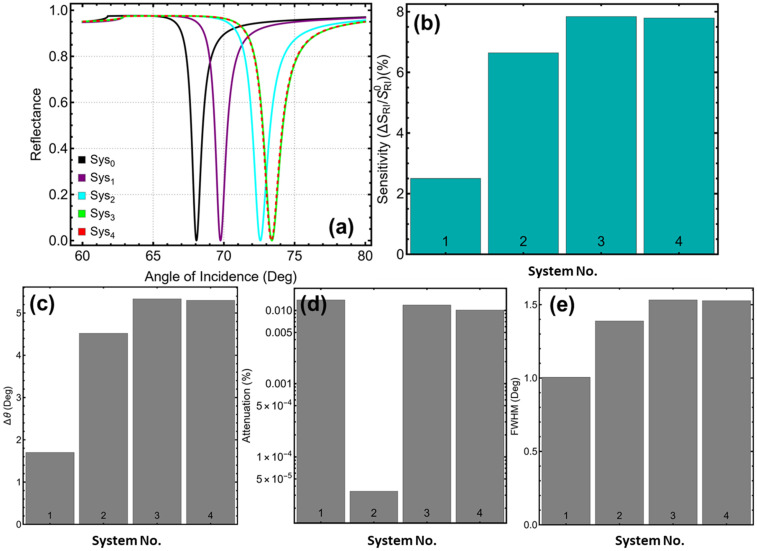
**Numerical modeling**. Performance comparison of SPR systems: (**a**) SPR response curves as a function of the incidence angle, (**b**) sensitivity enhancement relative to the baseline system (Sys_0_, black curve), (**c**) angular shift upon detection of a cancer sample, (**d**) attenuation percentage, and (**e**) full width at half maximum (FWHM) for each system.

The optical response of the four multilayer systems (Sys_1_ to Sys_4_) is assessed based on their angular reflectance behavior and extracted metrics, including SPR peak position, sensitivity enhancement relative to the baseline system (Sys_0_), angular shift ∆θ, attenuation, and FWHM (Figure 2). As stated, these metrics are derived from simulated reflectance curves obtained using the transfer matrix method, assuming an incident wavelength of 633 nm and refractive index variation from 1.335 to 1.349 in the sensing medium.

Therefore, the reflectance spectra as a function of incidence angle are shown in Figure 2a. The reference system, Sys_0_, exhibits a resonance dip at 68.12°, serving as the unperturbed baseline. Upon introducing additional layers and perturbations in the external medium, the resonance angles and curve shapes shift accordingly. In particular, Sys_1_ yields a resonance angle of 69.76° (Figure 2a) and registers a modest sensitivity enhancement of 2.50% (Figure 2b), with an angular shift ∆θ of 1.70° (Figure 2c). The attenuation reaches 0.014% (Figure 2d), while the FWHM narrows to 1.004° (Figure 2e), the smallest among all configurations considered.

On the other hand, Sys_2_, which incorporates a Si_3_N_4_ layer between Ag and the sensing medium, shows a marked performance improvement. The resonance peak shifts to 72.58°, sensitivity enhancement rises to 6.64%, and ∆θ increases to 4.52°. Notably, the system exhibits no attenuation (0.000%), indicating a well-defined resonance condition. The FWHM, however, widens to 1.389°, reflecting broader resonance. Sys_3_ introduces BP above the Si_3_N_4_ layer, producing one of the most pronounced shifts, with a resonance angle at 73.39°, a sensitivity enhancement of 7.84%, and ∆θ of 5.34°. Attenuation remains low at 0.012%, and the FWHM further increases to 1.532°. Sys_4_, which reverses the order of BP and Si_3_N_4_ relative to Sys_3_, yields comparable metrics: resonance at 73.36°, sensitivity enhancement of 7.79%, ∆θ of 5.30°, attenuation of 0.010%, and a FWHM of 1.526°. This suggests that both layer arrangements maintain efficient coupling and field interaction.

While all configurations will be examined in detail throughout this work, preliminary observations indicate that the inclusion of both Si_3_N_4_ and BP significantly improves sensitivity and angular resolution. These enhancements occur without inducing substantial attenuation, validating the physical viability of the multilayer designs under theoretical assumptions. The use of refractive index variations to simulate the sensing environment is consistent with numerical SPR modeling practices and is not intended to mimic the full complexity of biological analytes.

### 3.2. Metal Optimization

**Figure 3:**
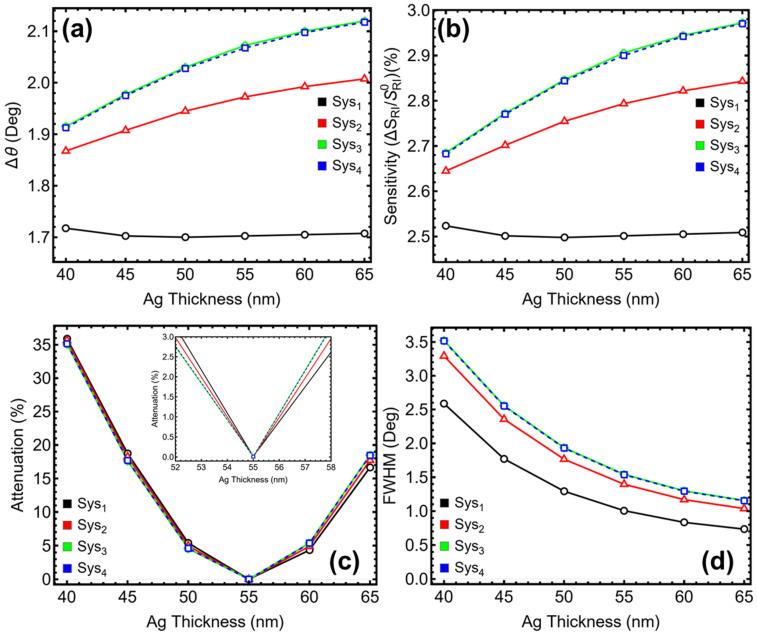
**Numerical modeling**. Performance metrics of the proposed sensors (Sys_1_–Sys_4_) as a function of silver layer thickness (40–65 nm): (**a**) angular shift, (**b**) sensitivity enhancement, (**c**) attenuation percentage, and (**d**) full width at half maximum (FWHM).

Silver is widely used as the plasmonic material in SPR platforms due to its favorable dielectric properties in the visible range. In conventional Kretschmann-based configurations operating at 633 nm, a thickness of 55.00 nm is commonly reported as optimal for efficient excitation of surface plasmon waves. However, the inclusion of additional dielectric and 2D materials—such as Si_3_N_4_ and black phosphorus—modifies the electromagnetic field distribution and resonance characteristics. For this reason, the silver layer thickness was systematically varied from 40.00 nm to 65.00 nm in steps of 5.00 nm to evaluate whether the standard value remains valid under the modified geometries of systems Sys_1_ through Sys_4_. The metrics evaluated include angular shift (∆θ) (Figure 3a), sensitivity enhancement relative to a reference system (Figure 3b), attenuation (Figure 3c), and FWHM (Figure 3c).

In Sys_1_, which lacks any enhancement layer, the angular shift at 55.00 nm is 1.70°, and the sensitivity enhancement is 2.50%. Attenuation is reduced to 0.01%, and the FWHM is narrowed to 1.01°, compared to 2.59° at 40.00 nm. Although other thicknesses show comparable ∆θ, they are associated with higher attenuation or broader resonance profiles. In Sys_2_, which includes a Si_3_N_4_ layer, the resonance behavior improves, and the optimal balance occurs again at 55.00 nm. At this value, ∆θ reaches 1.97°, sensitivity enhancement is 2.79%, and attenuation reaches a minimum of 0.00%, confirming optimal field coupling. The FWHM is 1.40°, which, while not the lowest observed, remains acceptably narrow given the low-loss condition. At higher thicknesses, marginal gains in angular shift and sensitivity are observed, but attenuation increases beyond 4.90%, making these configurations less favorable for precise refractive index sensing.

On the other hand, for Sys_3_ and Sys_4_, both of which incorporate Si_3_N_4_ and black phosphorus, the trends remain consistent. At 55.00 nm, Sys_3_ exhibits an angular shift of 2.07°, a sensitivity enhancement of 2.91%, and an attenuation of just 0.01%, with a corresponding FWHM of 1.54°. Sys_4_ shows nearly identical performance at the same thickness, with ∆θ = 2.07°, sensitivity enhancement = 2.90%, attenuation = 0.01%, and FWHM = 1.54°. While a further increase to 60.00 or 65.00 nm yields slightly higher ∆θ and sensitivity, the gains are accompanied by a steep rise in attenuation (up to 5.40% in Sys_3_ and 5.36% in Sys_4_), which compromises the quality and interpretability of the resonance signal. Therefore, 55.00 nm is selected as the operating thickness for all systems in the remainder of this study.

### 3.3. Silicon Nitride Optimization

**Figure 4:**
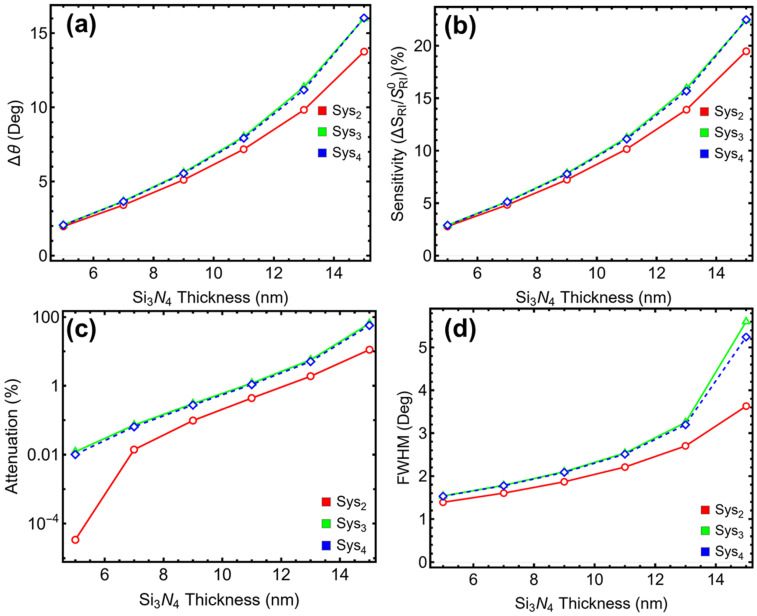
**Numerical modeling**. Performance metrics of the proposed sensors (Sys_2_–Sys_4_) as a function of silicon nitride layer thickness (5–15 nm): (**a**) angular shift, (**b**) sensitivity enhancement, (**c**) attenuation percentage, and (**d**) full width at half maximum (FWHM).

To assess the influence of the dielectric interlayer on the SPR sensor response, the thickness of the Si_3_N_4_ layer was varied between 5.00 nm and 15.00 nm in Sys_2_, Sys_3_, and Sys_4_, which are the only configurations including this component. The performance metrics—angular shift (∆θ) (Figure 4a), sensitivity enhancement (Figure 4b), attenuation (Figure 4c), and FWHM (Figure 4d)—are presented in Figure 4 and Table S5.

As the Si_3_N_4_ thickness increases, all three systems exhibit monotonic increases in ∆θ and sensitivity. For example, Sys_2_ shows growth in ∆θ from 1.97° at 5.00 nm to 13.76° at 15.00 nm, with sensitivity rising from 2.79% to 19.48%. Sys_3_ and Sys_4_ follow a similar trend, with ∆θ reaching 16.00° and sensitivity enhancements exceeding 22.40% at the maximum thickness evaluated. These gains, however, come at a cost; the attenuation percentage increases exponentially beyond 9.00 nm. In Sys_3_, for instance, attenuation rises from 0.30% at 9.00 nm to 5.66% at 13.00 nm, and sharply to 64.84% at 15.00 nm. Sys_4_ behaves comparably, with attenuation reaching 57.16% at 15.00 nm. This sharp increase in energy loss compromises resonance depth and reduces practical usability for refractive index sensing.

FWHM also deteriorates with increasing Si_3_N_4_ thickness. For Sys_2_, the FWHM increases from 1.39° at 5.00 nm to 3.63° at 15.00 nm. Sys_3_ and Sys_4_ show even greater degradation, with FWHM reaching 5.61° and 5.25°, respectively, at 15.00 nm. Broader resonances reduce the resolution and specificity of detection, particularly in applications that require precise angular discrimination. Based on these trade-offs, a Si_3_N_4_ thickness of 7.00 nm is selected as the optimal configuration across all three systems. At this value, the angular shift and sensitivity are substantially improved relative to the 5.00 nm baseline, without incurring the high attenuation and spectral broadening observed at larger thicknesses. The chosen Si_3_N_4_ thickness of 7.00 nm will therefore be used consistently in the following sections of this study to represent the dielectric enhancement layer in Sys_2_–Sys_4_.

### 3.4. Black Phosphorus Optimization

**Figure 5:**
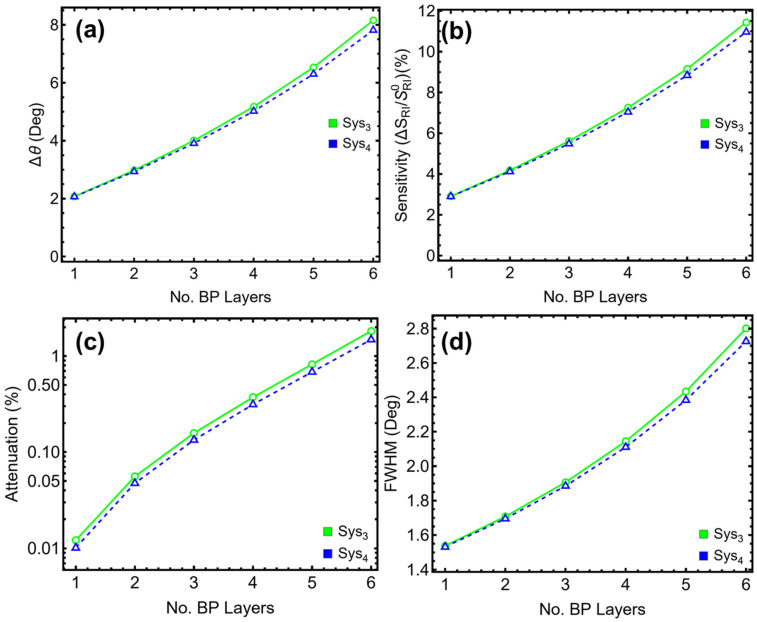
**Numerical modeling**. Performance metrics of the proposed sensors (Sys_3_ and Sys_4_) as a function of the number of BP layers (L1–L6): (**a**) angular shift, (**b**) sensitivity enhancement, (**c**) attenuation percentage, and (**d**) full width at half maximum (FWHM).

The number of black phosphorus (BP) layers plays a significant role in tuning the optical performance of multilayer SPR sensors due to their high in-plane anisotropy and strong plasmonic interaction. To quantify its impact, the BP layer count was varied from one to six layers (L1–L6) in Sys_3_ and Sys_4_, keeping all other parameters fixed. As shown in Figure 5 and detailed in Table S6, the inclusion of additional BP layers leads to progressive improvements in angular shift and sensitivity. In Sys_3_, the angular shift increases from 2.07° for a single layer to 8.16° at six layers (Figure 5a), with sensitivity enhancement rising from 2.91% to 11.44% (Figure 5b). A similar pattern is observed in Sys_4_, where the shift extends from 2.07° to 7.82°, and sensitivity enhancement reaches 10.96% at six layers. These enhancements are primarily attributed to the increased optical path length and field confinement introduced by the additional semiconducting material.

However, the improvements in ∆θ and sensitivity come at the cost of higher attenuation (Figure 5c) and broader resonance profiles (Figure 5d). In Sys_3_, attenuation grows from 0.01% at one layer to 1.83% at six layers, while the FWHM increases from 1.54° to 2.80°. Sys_4_ follows a nearly identical trend, with attenuation rising from 0.01% to 1.49% and FWHM expanding from 1.53° to 2.72° across the same range. Although these losses remain manageable up to four layers, beyond this point, the degradation in spectral sharpness becomes increasingly pronounced.

To achieve a suitable trade-off between enhanced sensitivity and acceptable spectral resolution, two BP layers were selected as the optimal configuration for both Sys_3_ and Sys_4_. At this thickness, Sys_3_ exhibits an angular shift of 2.98°, a sensitivity enhancement of 4.18%, attenuation of 0.06%, and FWHM of 1.71°. Sys_4_ produces similar values, with ∆θ = 2.94°, sensitivity enhancement = 4.12%, attenuation = 0.05%, and FWHM = 1.69°. These results reflect a configuration where performance is substantially improved over the single-layer case, while avoiding the resonance broadening and energy losses associated with thicker BP stacks.

To emphasize, the selected two-layer structure enables the BP film to enhance the local electromagnetic field without overwhelming the resonance conditions of the underlying Ag/Si_3_N_4_ interface. This makes it suitable for applications requiring improved detection sensitivity without compromising signal quality. Accordingly, two layers of BP are used in all subsequent simulations for Sys_3_ and Sys_4_ throughout this study.

Then, the final configurations of the four multilayer systems investigated in this work are summarized in Table S7. Each material’s refractive index and optimized thickness are listed for the respective structures. These values were obtained through parametric modeling aimed at balancing sensitivity, angular resolution, attenuation, and spectral sharpness. Notably, the Ag layer is fixed at 55.00 nm across all systems, while Si_3_N_4_ and BP thicknesses are adjusted only in the configurations where they are present. For black phosphorus, the thickness is indicated as 0.53 × L, with L representing the number of atomic layers, and two layers selected as optimal for Sys_3_ and Sys_4_.

Table S8 provides literature-based refractive index values associated with several biological cancer samples before and after the presence of specific conditions. These values are drawn from previously published experimental and theoretical studies [28,29,30,31,32,33]. The reported changes (Δn) typically range between 0.014 and 0.024, depending on the sample type and experimental context. It is important to note that while these values are often used in modeling refractive index changes within SPR sensors, they do not capture the full optical complexity of biological specimens. The simulations in this work therefore use refractive index variations in a generalized form to represent simplified perturbations in the sensing environment, without directly modeling or inferring biological states or detection.

### 3.5. Potential Applications for Cancer Detection

**Figure 6:**
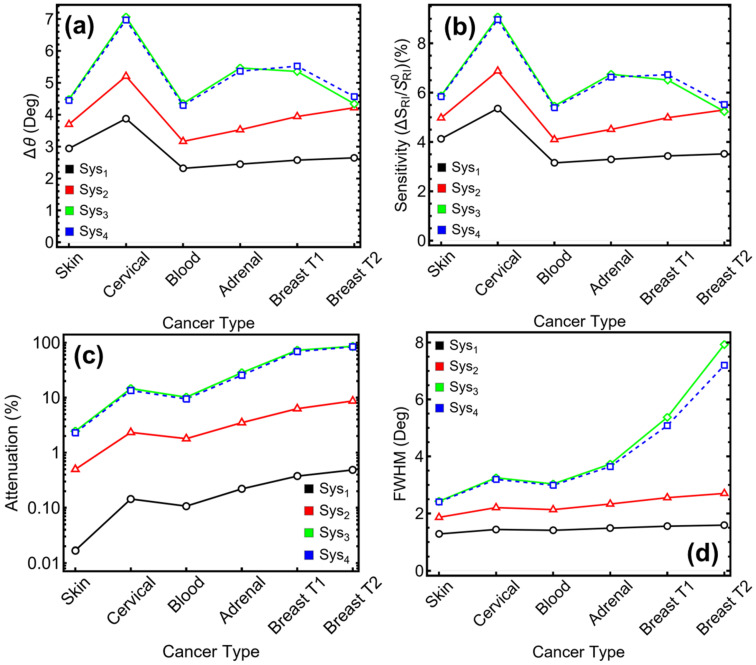
**Numerical modeling**. Performance metrics of the optimized sensors (Sys_1_–Sys_4_) as a function of different cancer types (Skin, Cervical, Blood, Adrenal, Breast T1 and T2): (**a**) angular shift, (**b**) sensitivity enhancement, (**c**) attenuation percentage, and (**d**) full width at half maximum (FWHM).

The final performance evaluation of the optimized SPR systems was conducted by applying refractive index perturbations that correspond to values found in prior theoretical and experimental literature for different biological environments. These variations are not intended to represent full biological behavior but serve to test the sensor response under realistic optical index shifts. Table S9 and Figure 6 summarize the behavior of Sys_1_ through Sys_4_ across six perturbation scenarios, labeled by their reference to biological samples for clarity: skin [28], cervical [29], blood [30], adrenal [31], breast T1 [32], and breast T2 [33]. In terms of angular shift (∆θ) (Figure 6a), Sys_1_ exhibits the lowest response across all perturbations, with values ranging from 2.32° (blood) to 3.88° (cervical). Sensitivity enhancement for this system remains below 5.40% across all cases (Figure 6b), with attenuation below 0.49% (Figure 6c) and FWHM ranging from 1.28° to 1.59° (Figure 6d). This confirms the limited response of the baseline silver-only configuration, consistent with its simpler optical architecture.

Sys_2_ introduces a 7.00 nm Si_3_N_4_ layer, resulting in a marked improvement in all metrics. Angular shifts increase to a range of 3.16°–5.21°, with sensitivity enhancements reaching 6.88% in the cervical case. However, these gains are accompanied by higher attenuation values, from 0.50% to 8.69%, and broader FWHM, increasing from 1.86° (skin) to 2.70° (breast T2). This trade-off reflects the increased field interaction introduced by the dielectric layer, which simultaneously enhances sensitivity while introducing additional optical loss.

The addition of black phosphorus in Sys_3_ amplifies the response even further. Angular shifts reach up to 7.06° for cervical perturbations, and sensitivity enhancement peaks at 9.07%. Across all perturbation cases, the sensitivity remains above 5.23%, indicating consistent responsiveness to refractive index changes. Nevertheless, this improved sensitivity leads to notable increases in attenuation, from 2.45% (skin) to 85.66% (breast T2). FWHM values follow a similar trend, widening from 2.43° to 7.93°. These results demonstrate the potential of BP-enhanced architectures to yield high angular resolution and sensitivity, though the resonance sharpness is increasingly compromised under larger perturbations.

Sys_4_, which reverses the position of BP and Si_3_N_4_, shows closely aligned trends to Sys_3_. Angular shifts vary from 4.29° (blood) to 6.97° (cervical), while sensitivity enhancement spans 5.39% to 8.96%. The attenuation values are slightly lower than in Sys_3_ at moderate perturbations but still escalate significantly at higher index shifts, with values ranging from 2.30% to 83.96%. FWHM increases from 2.41° (skin) to 7.20° (breast T2), highlighting the resonance broadening as a common limitation when strong optical confinement is achieved via multiple enhancement layers.

Across all systems, the cervical perturbation case—associated with a refractive index change from 1.368 to 1.392—triggers the most pronounced angular shift and sensitivity in Sys_3_ and Sys_4_. These results suggest that perturbations in this range are optimal for evaluating sensor performance within the dynamic operating window of multilayer SPR systems. Despite the significant improvements observed in Sys_3_ and Sys_4_, the rapid increase in attenuation and FWHM at higher index contrast presents a challenge for maintaining precise angular resolution, which must be considered in future design iterations.

To remark, while Sys_3_ and Sys_4_ demonstrate the highest sensitivity and angular shifts under all refractive index perturbations considered, they also exhibit increased energy loss and spectral broadening, particularly in scenarios with larger ∆n. Sys_2_ offers a more conservative trade-off with improved sensitivity over the baseline while maintaining more stable resonance characteristics. Sys_1_, though limited in response, remains the most stable configuration in terms of spectral sharpness and minimal attenuation. These insights highlight the importance of tailoring multilayer sensor architectures according to the specific refractive index range expected in the target sensing scenario, emphasizing performance tuning over universal optimization.

### 3.6. Performance Metrics of the Biosensor

**Figure 7:**
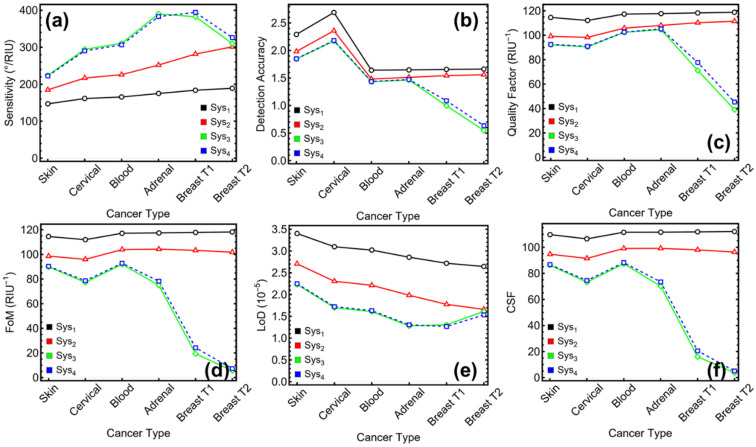
**Numerical modeling**. Performance metrics of the optimized sensors (Sys_1_–Sys_4_) for the detection of different cancer types: (**a**) sensitivity to refractive index variation, (**b**) detection accuracy (DA), (**c**) quality factor (QF), (**d**) figure of merit (FoM), (**e**) limit of detection (LoD), and (**f**) comprehensive sensitivity factor (CSF).

The evaluation of biosensor performance across Sys_1_ to Sys_4_ using multiple quantitative metrics reveals a nuanced trade-off between sensitivity and signal resolution. As shown in Table 1 and Figure 7, sensitivity (°/RIU) (Figure 7a) increases with the incorporation of enhancement layers, peaking in Sys_3_ and Sys_4_. In particular, Sys_3_ reaches a maximum sensitivity of 382.50°/RIU for the Breast T1 case, while Sys_4_ records 394.46°/RIU under the same perturbation. These values are significantly higher than the baseline Sys_1_ (183.92°/RIU) and confirm the enhanced responsiveness of hybrid configurations incorporating Si_3_N_4_ and BP.

Despite these high sensitivity values, other metrics reflect the consequences of increased optical complexity. For example, the quality factor (QF) (Figure 7b), defined as the sensitivity-to-FWHM ratio, decreases in Sys_3_ and Sys_4_ under stronger index shifts. For Breast T2, Sys_3_ yields 39.09 RIU^−1^ and Sys_4_ drops to 45.28 RIU^−1^, whereas Sys_1_ maintains a QF of 118.78 RIU^−1^. This inverse trend stems from broader resonance widths in Sys_3_ and Sys_4_, which, although expected due to stronger field confinement, diminish the angular selectivity.

Detection accuracy (DA) (Figure 7c) also declines at higher refractive index shifts for Sys_3_ and Sys_4_. While moderate in the skin and cervical cases (e.g., DA = 2.17 for Sys_3_ and 2.18 for Sys_4_ in cervical), the values fall sharply under stronger perturbations, such as 0.54 and 0.63 for Breast T2, respectively. Sys_1_, by contrast, remains relatively stable, maintaining DA values between 1.64 and 2.69. However, the overall sensitivity of Sys_1_ is lower, limiting its applicability in detecting smaller changes.

When comparing the figure of merit (FoM) (Figure 7d), Sys_1_ again shows consistent and high values across all cases, ranging from 111.95 to 118.20 RIU^−1^, due to its narrower FWHM. Sys_2_ exhibits slightly lower but still stable FoM values (96.00 to 103.90 RIU^−1^), making it the most balanced system for minimizing energy loss while improving detection sensitivity. In contrast, FoM in Sys_3_ and Sys_4_ falls significantly as refractive index perturbation increases, reaching 5.60 RIU^−1^ and 7.26 RIU^−1^ for Breast T2, respectively. These values, although lower, must be interpreted in light of the high sensitivity values achieved.

The limit of detection (LoD) (Figure 7e) follows a similar pattern. Sys_3_ and Sys_4_ show LoD values as low as 1.26 × 10^−5^ RIU (Sys_4_, Breast T1), and 1.30 × 10^−5^ RIU (Sys_3_, Adrenal), demonstrating their ability to detect small refractive index changes under favorable conditions. However, the LoD increases in cases where spectral broadening dominates (e.g., Breast T2), reaching 1.61 × 10^−5^ RIU for Sys_3_ and 1.53 × 10^−5^ RIU for Sys_4_. In contrast, Sys_1_ shows a higher but more stable LoD across all cases, with values between 2.64 and 3.40 × 10^−5^ RIU.

Finally, the comprehensive sensitivity factor (CSF) (Figure 7f), which accounts for angular sensitivity, spectral sharpness, and energy losses, highlights the multidimensional performance of each system. Sys_1_ records the highest CSF values overall, peaking at 111.55 for Blood and 112.10 for Breast T2. However, this performance is mainly due to its narrower FWHM rather than high angular sensitivity. Sys_3_ and Sys_4_, although exhibiting lower CSF in extreme perturbation scenarios (e.g., 3.73 for Sys_3_, Breast T2), provide superior CSF values in moderate-index shifts (e.g., 86.36 for Skin in Sys_3_ and 86.72 for Sys_4_), where sensitivity is enhanced without significant deterioration in other metrics.

Then, Sys_1_ and Sys_2_ show strong performance in stability-related metrics such as QF, DA, FoM, and LoD, making them advantageous in applications requiring high spectral resolution and low optical loss. However, Sys_3_ and Sys_4_ demonstrate outstanding angular sensitivity and detection potential under small-to-moderate index shifts, which may be particularly useful for applications demanding heightened responsiveness. The observed trade-offs underscore the need to select or design sensor architectures based on the specific operating window and the desired balance between sensitivity and spectral precision.

### 3.7. Literature Comparison

The sensitivity performance of the proposed configurations is further contextualized in Table 2 by comparing the results obtained for the Breast T1 perturbation case with representative designs reported in previous literature. A configuration using PtSe_2_ as a plasmonic enhancer over a BK7-Ag platform reached a sensitivity of 228.57°/RIU, as reported in [34], while a BK7-Au-Ag-AlN hybrid system demonstrated 385.00°/RIU under similar index contrast conditions [24]. These configurations offer valuable benchmarks in the development of high-sensitivity SPR biosensors through the incorporation of transition metal dichalcogenides and dielectric tuning.

In comparison, both multilayer configurations proposed in this study—BK7-Ag-Si_3_N_4_-BP (Sys_3_) and BK7-Ag-BP-Si_3_N_4_ (Sys_4_)—exhibit sensitivity values of 382.50°/RIU and 394.46°/RIU, respectively, for the Breast T1 case. These results place both designs at or above the upper end of the current reported performance. The improvement is attributed to the cooperative enhancement provided by Si_3_N_4_, which increases field confinement, and black phosphorus, which contributes to strong in-plane coupling and optical anisotropy.

This outcome supports the potential utility of the Ag/BP/Si_3_N_4_ and Ag/Si_3_N_4_/BP layer sequences for refractive index sensing scenarios where sensitivity maximization is a primary objective.

## 4. Conclusions

In summary, this study developed a numerical modeling framework to evaluate and optimize the performance of multilayer SPR sensors based on the Kretschmann configuration. Four configurations (Sys_1_–Sys_4_), incorporating combinations of silver (Ag), silicon nitride (Si_3_N_4_), and black phosphorus (BP), were examined under refractive index variations in the range of 1.360–1.401. The transfer matrix method was employed to compute reflectance behavior and extract performance metrics including angular shift, sensitivity enhancement, FWHM, attenuation, FoM, QF, DA, LoD, and CSF.

A complete parametric optimization strategy was carried out for the thickness of Ag and Si_3_N_4_ layers, as well as for the number of BP layers, to identify configurations that offer a favorable trade-off between sensitivity and spectral resolution. The optimal structure was found to be a hybrid multilayer sensor integrating both Si_3_N_4_ and two BP layers, which exhibited the highest angular sensitivity (up to 394.46°/RIU), along with detection limits on the order of 10^−5^ RIU. These enhancements were supported by consistent improvements across secondary metrics in moderate perturbation scenarios.

Importantly, the results showed that sensitivity maximization often leads to increased attenuation and broader resonance peaks, reducing spectral precision. In contrast, simpler configurations (Sys_1_ and Sys_2_) yielded more stable values for FoM, QF, and CSF, especially under large refractive index shifts. This underscores the value of using a multi-parameter evaluation approach when designing SPR sensors, as reliance on sensitivity alone can obscure critical performance limitations.

All results presented are based exclusively on numerical simulations assuming idealized optical conditions. As such, the refractive index changes used here serve as simplified proxies for environmental perturbations, not biological specificity. Nonetheless, the modeling strategy, optimization workflow, and performance analysis framework established in this study offer a valuable reference for the design and pre-evaluation of multilayer SPR sensors in theoretical and applied research contexts.

## Figures and Tables

**Table 1:** **Numerical modeling**. Performance metrics of the optimized SPR biosensors for different cancer types.

Modeled RI Case	*S* (°/RIU)	DA	QF (*RIU*^−1^)	FoM (*RIU*^−1^)	LoD (10^−5^)	CSF
Sys_1_						
Skin	147.00	2.29	114.57	114.55	3.40	109.75
Cervical	161.45	2.69	112.11	111.95	3.09	106.47
Blood	165.53	1.64	117.31	117.19	3.02	111.55
Adrenal	175.00	1.65	117.72	117.46	2.85	111.59
Breast T1	183.92	1.66	118.30	117.86	2.71	111.83
Breast T2	189.10	1.66	118.78	118.20	2.64	112.10
**Sys_2_**						
Skin	184.87	1.98	99.11	98.61	2.70	94.69
Cervical	216.87	2.35	98.29	96.00	2.30	91.48
Blood	225.89	1.48	105.80	103.90	2.21	99.12
Adrenal	251.96	1.51	108.03	104.24	1.98	99.18
Breast T1	281.60	1.54	110.25	103.31	1.77	98.02
Breast T2	301.25	1.56	111.45	101.76	1.65	96.35
**Sys_3_**						
Skin	224.00	1.84	92.22	89.96	2.23	86.36
Cervical	294.27	2.17	90.51	77.28	1.69	73.18
Blood	310.71	1.43	102.41	91.98	1.60	87.42
Adrenal	390.35	1.46	104.64	74.98	1.28	70.14
Breast T1	382.5	0.99	71.20	19.39	1.30	16.02
Breast T2	310.00	0.54	39.09	5.60	1.61	3.73
**Sys_4_**						
Skin	222.37	1.84	92.45	90.33	2.24	86.72
Cervical	290.31	2.18	90.88	78.63	1.72	74.52
Blood	306.42	1.43	102.54	92.84	1.63	88.27
Adrenal	383.03	1.47	105.15	78.24	1.30	73.38
Breast T1	394.46	1.08	77.68	24.21	1.26	20.53
Breast T2	326.07	0.63	45.28	7.26	1.53	5.09

**Table 2:** Comparison with state-of-the-art biosensor records.

Configuration	S (°/RIU)	Refs.
BK7-Ag-PtSe_2_-Breast T1	228.57	[34]
BK7-Au-Ag-AlN-Breast T1	385.00	[24]
BK7-Ag-Si_3_N_4_-BP-Breast T1	382.50	This work
BK7-Ag-BP-Si_3_N_4_-Breast T1	394.46	This work

## Data Availability

The original contributions presented in the study are included in the article/Supplementary Materials, further inquiries can be directed to the corresponding author.

## Supplementary Materials

The following supporting information can be downloaded at https://www.mdpi.com/article/10.3390/s25092685/s1, Table S1. Systems under study. Table S2. Initial parameters of the main components of the SPR Biosensor. Table S3. Metrics of the different systems under consideration. Table S4. Metrics of the different systems under consideration by varying the silver thickness. Table S5. Metrics of the different systems under consideration by varying the silicon nitride thickness. Table S6. Metrics of the different systems under consideration by varying the number of BP layers. Table S7. Optimized parameters of the different systems considered in this study. Table S8. Refractive index values for the different cancer types. The values of RI are reported before (normal) and after the presence. Table S9. Metrics of the different optimized systems for different cancer types.
