# Supplementary material for: Advanced SPR-Based Biosensors for Potential Use in Cancer Detection: A Theoretical Approach
Source: Sensors (Basel). 2025 Apr 24;25(9):2685. doi: 10.3390/s25092685 (PMC12074272; doi:10.3390/s25092685)
Supplement: Supplementary file 1 [file sensors-25-02685-s001.zip › sensors-3596564-supplementary.pdf]

SUPPLEMENTARY INFORMATION: Advanced SPR-Based Biosensor for Potential Use in Cancer Detection: A Theoretical Approach

Talia Tene[1,*], Fabian Arias Arias[2,3], Darío Fernando Guamán-Lozada[3], María Augusta Guadalupe Alcoser[3], Lala Gahramanli[4,5], Cristian Vacacela Gomez[6], Stefano Bellucci[6]

[1]Department of Chemistry, Universidad Técnica Particular de Loja, Loja 110160, Ecuador
[2]Department of Chemistry and Chemical Technologies, University of Calabria, Via P. Bucci, Cubo 9 15D, 87036 Arcavacata di Rende, Italy
[3]Facultad de Ciencias, Escuela Superior Politécnica de Chimborazo (ESPOCH), Riobamba, 060155, Ecuador
[4]Nano Research Laboratory, Excellent Center, Baku State University, Baku, Azerbaijan
[5]Chemical Physics of Nanomaterials, Physics Department, Baku State University, Baku, Azerbaijan
[6]INFN-Laboratori Nazionali di Frascati, Via E. Fermi 54, 00044 Frascati, Italy

* Correspondence:
T. Tene: tbtene@utpl.edu.ec

Supplementary Tables

Table S1. Systems under study

Sys No.	Code	Full Name	Nick Name
0	Sys_0	Prism/Silver/PBS Medium	P/Ag/M_{PBS}
1	Sys_1	Prism/Silver/Cancer Sample	P/Ag/M_{Cancer}
2	Sys_2	Prism/Silver/S_3N_4/Cancer Sample	P/Ag/SN/M_{Cancer}
3	Sys_3	Prism/Silver/S_3N_4/Black Phosphorus/Cancer Sample	P/Ag/SN/BP/M_{Cancer}
2	Sys_4	Prism/Silver/Black Phosphorus/S_3N_4/Cancer Sample	P/Ag/BP/SN/M_{Cancer}

Table S2. Initial parameters of the main components of the SPR Biosensor

Material	Refractive Index	Thickness (nm)	Ref.
BK-7 (P)	1.5151	---	[17]
Silver (Ag)	0.056253 + 4.2760 i	55.0	[26]
Si_3N_4 (SN)	2.0394	5.00	[13]
Black Phosphorus (BP)	3.5 + 0.01 i	0.53	[27]
PBS (M)	1.335	---	[16]
Cancer Sample (Δn)	1.349	---	[24]

Table S3. Metrics of the different systems under consideration

Sys No.	Code	SPR Peak position	Sensitivity Enhancement (%)	$\Delta\theta$ (Deg)	Attenuation (%)	FWHM
1	Sys_1	69.760	2.501	1.702	0.014	1.004
2	Sys_2	72.580	6.644	4.522	0.000	1.389
3	Sys_3	73.394	7.840	5.336	0.012	1.532
4	Sys_4	73.360	7.790	5.302	0.010	1.526

Table S4. Metrics of the different systems under consideration by varying the silver thickness

Sys No.	SPR Peak position	Sensitivity Enhancement (%)	$\Delta\theta$ (Deg)	Attenuation (%)	FWHM
**Sys_1**					
40	69.775	2.523	1.717	35.897	2.587
45	69.760	2.501	1.702	18.734	1.770
50	69.757	2.497	1.700	5.404	1.293
55	69.760	2.501	1.702	0.013	1.006
60	69.762	2.505	1.705	4.352	0.832
65	69.765	2.508	1.707	16.670	0.733
**Sys_2**					
40	72.475	2.644	1.867	35.660	3.293
45	72.515	2.701	1.907	18.245	2.356
50	72.552	2.754	1.945	4.972	1.765
55	72.580	2.793	1.972	0.000	1.396
60	72.600	2.821	1.992	4.928	1.168
65	72.615	2.843	2.007	17.723	1.037
**Sys_3**					
40	73.237	2.684	1.915	35.082	3.522
45	73.300	2.772	1.977	17.638	2.558
50	73.352	2.846	2.030	4.570	1.936
55	73.395	2.905	2.072	0.012	1.542
60	73.422	2.944	2.100	5.398	1.298
65	73.442	2.972	2.120	18.510	1.157
**Sys_4**					
40	73.205	2.682	1.912	35.138	3.515
45	73.267	2.770	1.975	17.691	2.551
50	73.320	2.843	2.027	4.603	1.930
55	73.360	2.900	2.067	0.010	1.536
60	73.390	2.942	2.097	5.360	1.293
65	73.410	2.970	2.117	18.449	1.152

Table S5. Metrics of the different systems under consideration by varying the silicon nitride thickness

Sys No.	SPR Peak position	Sensitivity Enhancement (%)	$\Delta\theta$ (Deg)	Attenuation (%)	FWHM
			**Sys_2**		
5	72.580	2.793	1.972	0.000	1.390
7	74.020	4.833	3.412	0.013	1.603
9	75.720	7.240	5.112	0.097	1.868
11	77.782	10.161	7.175	0.437	2.210
13	80.432	13.915	9.825	1.886	2.700
15	84.365	19.484	13.757	11.180	3.627
			**Sys_3**		
5	73.395	2.905	2.072	0.012	1.537
7	75.000	5.156	3.677	0.071	1.786
9	76.927	7.858	5.605	0.300	2.103
11	79.345	11.248	8.022	1.186	2.536
13	82.692	15.941	11.370	5.656	3.248
15	87.320	22.429	15.997	64.835	5.605
			**Sys_4**		
5	73.360	2.900	2.067	0.010	1.531
7	74.945	5.123	3.652	0.064	1.777
9	76.842	7.784	5.550	0.274	2.088
11	79.217	11.116	7.925	1.085	2.511
13	82.472	15.681	11.180	5.078	3.195
15	87.322	22.484	16.030	57.163	5.245

Table S6. Metrics of the different systems under consideration by varying the number of BP layers

Sys No.	SPR Peak position	Sensitivity Enhancement (%)	$\Delta\theta$ (Deg)	Attenuation (%)	FWHM
			**Sys_3**		
L1	73.395	2.905	2.072	0.012	1.537
L2	74.302	4.178	2.980	0.055	1.707
L3	75.327	5.615	4.005	0.157	1.906
L4	76.497	7.255	5.175	0.371	2.143
L5	77.855	9.159	6.532	0.821	2.433
L6	79.480	11.437	8.157	1.826	2.801
			**Sys_4**		
L1	73.360	2.900	2.067	0.010	1.531
L2	74.227	4.116	2.935	0.047	1.694
L3	75.202	5.484	3.910	0.133	1.884
L4	76.312	7.041	5.020	0.312	2.110
L5	77.592	8.836	6.300	0.682	2.383
L6	79.107	10.961	7.815	1.485	2.725

Table S7. Optimized parameters of the different systems considered in this study

**Material**	**Refractive Index (RI)**	**Thickness (nm)**
	**Sys_1**	
BK7 (P)	1.5151	---
Ag	0.056253 + 4.2760	55.0
	**Sys_2**	
BK7 (P)	1.5151	---
Ag	0.056253 + 4.2760	55.0
S_3N_4 (SN)	2.0394	7.0
	**Sys_3**	
BK7 (P)	1.5151	---
Ag	0.056253 + 4.2760	55.0
S_3N_4 (SN)	2.0394	7.0
Black Phosphorus (BP)	3.5 + 0.01 i	0.53*L (L=2)
	**Sys_4**	
BK7 (P)	1.5151	---
Ag	0.056253 + 4.2760	55.0
Black Phosphorus (BP)	3.5 + 0.01 i	0.53*L (L=2)
S_3N_4 (SN)	2.0394	7.0

Table S8. Refractive index values for the different cancer types. The values of RI are reported before (normal) and after the presence

**Cancer Type**	**RI Normal**	**RI Cancer Presence**	**Δn**	**Ref.**
Skin (Basal)	1.360	1.380	0.020	[28]
Cervical (HeLa)	1.368	1.392	0.024	[29]
Blood (Jurkat)	1.376	1.390	0.014	[30]
Adrenal (PC-12)	1.381	1.395	0.014	[31]
Breast T1 (MM-231)	1.385	1.399	0.014	[32]
Breast T2 (MCF-7)	1.387	1.401	0.014	[33]

Table S9. Metrics of the different optimized systems for different cancer types

Cancer Type	SPR Peak position	Sensitivity Enhancement (%)	$\Delta\theta$ (Deg)	Attenuation (%)	FWHM
			**Sys_1**		
Skin	74.142	4.129	2.940	0.016	1.283
Cervical	76.195	5.385	3.875	0.144	1.440
Blood	75.832	3.152	2.317	0.107	1.411
Adrenal	76.755	3.297	2.450	0.219	1.486
Breast T1	77.540	3.434	2.575	0.374	1.554
Breast T2	77.952	3.515	2.647	0.485	1.592
			**Sys_2**		
Skin	77.992	4.976	3.697	0.497	1.865
Cervical	80.867	6.879	5.205	2.329	2.206
Blood	80.332	4.098	3.162	1.791	2.135
Adrenal	81.735	4.510	3.527	3.502	2.332
Breast T1	83.045	4.984	3.942	6.293	2.554
Breast T2	83.795	5.299	4.217	8.694	2.703
			**Sys_3**		
Skin	80.730	5.875	4.480	2.451	2.428
Cervical	84.915	9.071	7.062	14.621	3.251
Blood	84.035	5.458	4.350	10.184	3.033
Adrenal	86.472	6.746	5.465	28.347	3.730
Breast T1	87.572	6.513	5.355	72.765	5.372
Breast T2	87.235	5.235	4.340	85.661	7.930
			**Sys_4**		
Skin	80.615	5.839	4.447	2.295	2.405
Cervical	84.727	8.960	6.967	13.475	3.194
Blood	83.870	5.390	4.290	9.454	2.988
Adrenal	86.252	6.629	5.362	25.587	3.642
Breast T1	87.607	6.727	5.522	68.832	5.077
Breast T2	87.315	5.516	4.565	83.958	7.199
